# Structure of reverse gyrase with a minimal latch that supports ATP-dependent positive supercoiling without specific interactions with the topoisomerase domain

**DOI:** 10.1107/S2059798323002565

**Published:** 2023-05-19

**Authors:** Vaibhav P. Mhaindarkar, René Rasche, Daniel Kümmel, Markus G. Rudolph, Dagmar Klostermeier

**Affiliations:** a Institute for Physical Chemistry, University of Muenster, Corrensstrasse 30, 48149 Muenster, Germany; b Institute for Biochemistry, University of Muenster, Corrensstrasse 36, 48149 Muenster, Germany; c Pharma Research and Early Development, Molecular Design and Chemical Biology, Hoffmann-La Roche, Grenzacherstrasse 124, 4070 Basel, Switzerland; University of Cambridge, United Kingdom

**Keywords:** reverse gyrases, helicases, topoisomerases, latch, positive DNA supercoiling, extremophiles, inter-domain communication

## Abstract

Reverse gyrase is the only topoisomerase that introduces positive supercoils into DNA in an ATP-dependent reaction. Positive DNA supercoiling becomes possible through the functional cooperation of the N-terminal helicase domain of reverse gyrase with its C-terminal type IA topoisomerase domain. This cooperation is mediated by a reverse-gyrase-specific insertion into the helicase domain termed the ‘latch’. The latch consists of a globular domain inserted at the top of a β-bulge loop that connects this globular part to the helicase domain. While the globular domain shows little conservation in sequence and length and is dispensable for DNA supercoiling, the β-bulge loop is required for supercoiling activity. It has previously been shown that the β-bulge loop constitutes a minimal latch that couples ATP-dependent processes in the helicase domain to DNA processing by the topoisomerase domain. Here, the crystal structure of *Thermotoga maritima* reverse gyrase with such a β-bulge loop as a minimal latch is reported. It is shown that the β-bulge loop supports ATP-dependent DNA supercoiling of reverse gyrase without engaging in specific interactions with the topoisomerase domain. When only a small latch or no latch is present, a helix in the nearby helicase domain of *T. maritima* reverse gyrase partially unfolds. Comparison of the sequences and predicted structures of latch regions in other reverse gyrases shows that neither sequence nor structure are decisive factors for latch functionality; instead, the decisive factors are likely to be electrostatics and plain steric bulk.

## Abstract 2

The latch domain in reverse gyrases mediates the cooperation of the helicase and topoisomerase domains. In *Thermotoga maritima* reverse gyrase this latch can be designed into a minimal β-bulge loop that has natural precedence in *Thermosipho africanus* reverse gyrase. The properties and functionalities of different latch domains across reverse gyrases are discussed.

## 1. Introduction

Topoisomerases are a class of essential enzymes that change the topology of DNA during replication, repair and transcription (reviewed in Vos *et al.*, 2011 ▸; Hirsch & Klostermeier, 2021 ▸; McKie *et al.*, 2021 ▸). The removal or introduction of supercoils, catenation/decatenation and knotting/unknotting are achieved by transiently cleaving the ribose-phosphate backbones of their DNA substrates. The catalytic residue responsible for DNA cleavage by topoisomerases is a tyrosine that forms a transient tyrosyl-phosphate ester with either the 5′-side or the 3′-side of the phosphoryl group (Liu & Wang, 1979 ▸; Champoux, 1981 ▸; Horowitz & Wang, 1987 ▸). Depending on whether one or both DNA strands are cleaved, topo­isomerases are classified into the type I or type II families. Type I enzymes change the supercoiling state of DNA either by allowing the uncleaved strand to pass through the gap (type IA) or by controlled rotation (swivelases, type IB). Similar to type IA enzymes (recently reviewed in Bizard & Hickson (2020 ▸), type II topoisomerases use strand passage for supercoiling. Religation of the phosphate backbone fixes the supercoiling state of the DNA. Interruption of the topo­isomerase reaction leads to DNA breakage and, if not repaired, to cell death. Therefore, topoisomerases are of pharmaceutical interest as both anticancer and antibiotic targets (Nitiss, 2009 ▸; Collin *et al.*, 2011 ▸; Wu *et al.*, 2013 ▸; Tse-Dinh, 2016 ▸).

Type II topoisomerases need the energy of ATP hydrolysis for both the introduction and the removal of supercoils (Goto & Wang, 1982 ▸). In contrast, type I topoisomerases do not bind ATP. Members of this topoisomerase family support the exergonic relaxation of positively and negatively supercoiled DNA, but cannot supercoil DNA (Dekker *et al.*, 2002 ▸). The only exception is the introduction of positive supercoils by a special enzyme known as reverse gyrase. Reverse gyrase is a bacterial/archaeal type IA DNA topoisomerase that catalyzes the ATP-dependent introduction of positive supercoils into DNA (Kikuchi & Asai, 1984 ▸; Nakasu & Kikuchi, 1985 ▸; Kikuchi *et al.*, 1986 ▸; Shibata *et al.*, 1987 ▸; for a review, see Lulchev & Klostermeier, 2014 ▸). The presence of this enzyme is a hallmark of thermophilicity (Forterre, 2002 ▸). Reverse gyrase protects DNA against thermal denaturation and damage, and has been implicated in DNA repair (Han *et al.*, 2017 ▸; Napoli *et al.*, 2004 ▸; Valenti *et al.*, 2006 ▸, 2009 ▸; Perugino *et al.*, 2009 ▸; Kampmann & Stock, 2004 ▸; Hsieh & Plank, 2006 ▸).

**Figure 1:**
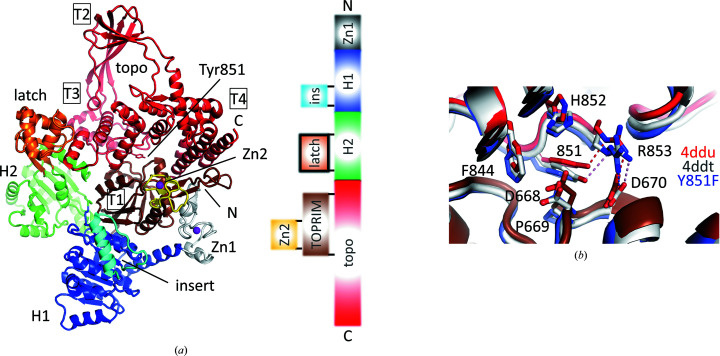
Domain organization and active site in reverse gyrases. (*a*) Structure of *T. maritima* wild-type reverse gyrase (PDB entry 4ddu) colored according to the subdomains present in reverse gyrases. The right-hand side maps the domains onto the sequence, showing that some subdomains are inserts in larger domains. The H1 and H2 domains are RecA-like folds that constitute the helicase domain. The latch (orange) is an insert in the H2 domain and ‘latches’ against the topoisomerase domain (red). It is a short, two-stranded β-sheet with a globular, helical domain inserted at its tip. The topoisomerase domain harbors the catalytic tyrosine (Tyr851 in *T. maritima*). It is subdivided into four regions termed T1–T4 (boxed). (*b*) The Y851F variant (blue) has little effect on the structure of the active site. The plasticity between the wild-type structures PDB entries 4ddu (red) and 4ddt (gray) is on the same scale as that on comparison with the Y851F variant. Hence, the Y851F variant structure can be included in the group of structures for an unbiased comparison with rgyr_minlatch.

The general architecture of reverse gyrase has become evident from crystal structures of the enzymes from *Archaeoglobus fulgidus* (Rodríguez & Stock, 2002 ▸) and *Thermotoga maritima* (Rudolph *et al.*, 2013 ▸). Both enzymes are large (>120 kDa) monomeric entities that consist of a helicase domain containing the ATP-binding site and a type IA topoisomerase domain (Fig. 1 ▸ *a*) fused on the same poly­peptide chain. Their overall shape resembles a padlock, with the helicase and topoisomerase domains loosely connected by a ‘latch’ (see below). The helicase domain consists of two RecA-like domains (termed H1 and H2) that carry degenerate versions of conserved sequence motifs typical of the superfamily 2 helicase family (Ganguly *et al.*, 2011 ▸, 2013 ▸; del Toro Duany & Klostermeier, 2011 ▸; del Toro Duany *et al.*, 2008 ▸; Rodríguez, 2002 ▸). Mutations in these motifs abrogate reverse gyrase activity (Bouthier de la Tour *et al.*, 2008 ▸). The helicase domain of *T. maritima* reverse gyrase on its own switches between an open conformation in the absence of substrates and a closed conformation when ATP and DNA are bound (del Toro Duany & Klostermeier, 2011 ▸). This conformational change is linked to switching of the helicase domain from a high-affinity state for single-stranded DNA (ssDNA) to a high-affinity state for double-stranded DNA (dsDNA) (del Toro Duany *et al.*, 2008 ▸; del Toro Duany & Klostermeier, 2011 ▸). The isolated helicase domain of *T. maritima* reverse gyrase, as well as the full-length enzyme, can unwind short DNA duplex regions in an ATP-dependent reaction (Ganguly *et al.*, 2013 ▸). The topoisomerase domain consists of four subdomains, termed T1 to T4. It contains the catalytic tyrosine residue that acts as a nucleophile for DNA cleavage. For the convenience of biochemical studies, this residue is often mutated to phenylalanine, which renders reverse gyrase inactive but still able to bind its substrates (Li *et al.*, 2011 ▸; del Toro Duany *et al.*, 2008 ▸) (Fig. 1 ▸ *b*).

The two parts of reverse gyrase, the helicase domain and the topoisomerase domain, are structurally connected by the latch, an insertion in the helicase subdomain H2. The pictorial term ‘latch’ was coined based on the position of this element at the interface of the two domains, where it engages in contacts with the topoisomerase T3 domain that stabilize the padlock-like structure (Rodríguez & Stock, 2002 ▸). This location suggested a functional role in regulating the transient opening of the topoisomerase domain during catalysis (Rodríguez & Stock, 2002 ▸). In the crystal structures of reverse gyrases from *T. maritima* and *A. fulgidus*, the latch consists of a globular domain inserted at the tip of a short two-stranded antiparallel β-sheet emerging from the H2 domain (Rodríguez & Stock, 2002 ▸; Rudolph *et al.*, 2013 ▸). The latch couples the activity of the helicase domain to strand passage (del Toro Duany *et al.*, 2014 ▸; Ganguly *et al.*, 2011 ▸; Rodríguez, 2002 ▸, 2003 ▸). It has been suggested to prevent ATP-independent DNA relaxation by the topoisomerase domain (Rodríguez & Stock, 2002 ▸). This is indeed the case for *A. fulgidus* reverse gyrase, which catalyzes ATP-independent DNA relaxation (Rodríguez & Stock, 2002 ▸), but not for the *T. maritima* enzyme (Jungblut & Klostermeier, 2007 ▸). While the sequence of the two β-strands connecting the globular part of the latch to H2 is rather conserved among reverse gyrases, the globular domain shows little conservation in length or in sequence (Collin *et al.*, 2020 ▸; Lulchev & Klostermeier, 2014 ▸). We previously showed that a reverse gyrase in which the globular domain of the latch was deleted by connecting these two β-strands directly by a loop still catalyzes DNA supercoiling (Collin *et al.*, 2020 ▸). Such a β-bulge loop (Milner-White, 1987 ▸) thus constitutes a minimal latch that enables coupling between the domains and supports basal DNA supercoiling activity. Here, we report the crystal structure of *T. maritima* reverse gyrase containing this minimal latch at 2.9 Å resolution. We show that despite functioning as a latch, the β-bulge loop does not engage in specific interactions with the topoisomerase domain, in contrast to the larger latch domains that are present in most other reverse gyrases. The minimal latch thus seems to simply act by its presence and possibly by its electrostatic characteristics.

## 2. Materials and methods

### 2.1. Protein purification

*T. maritima* reverse gyrase with a minimal latch (rgyr_minlatch), lacking amino acids 395–455 and fused to a C-terminal TEV-cleavable His_6_ tag, was produced recombinantly in *Escherichia coli* Rosetta (DE3) and purified as described previously (Collin *et al.*, 2020 ▸). Briefly, the cells were disrupted in 50 m*M* Tris–HCl pH 7.5, 800 m*M* NaCl, 20 m*M* imidazole, 2 m*M* β-mercaptoethanol using a microfluidizer. Reverse gyrase was purified to homogeneity by chromatography on Ni^2+^–NTA Sepharose, followed by cleavage of the His_6_ tag with TEV protease, removal of uncleaved fusion protein and the tag by Ni^2+^–NTA Sepharose, removal of DNA by chromatography on Q Sepharose and a final size-exclusion step on an S200 column in 50 m*M* Tris–HCl pH 7.5, 500 m*M* NaCl, 10 m*M* MgCl_2_, 100 µ*M* zinc acetate, 2 m*M* β-mercaptoethanol. Purification of the Y851F variant (rgyr_Y851F) followed the same protocol.

### 2.2. Crystallization and structure determination

Crystals of rgyr_minlatch and rgyr_Y851F were obtained at 295 K in the sitting-drop vapor-diffusion format by mixing 0.2 µl each of enzyme and reservoir solution. Rgyr_minlatch (at 54 µ*M*) crystallized from 20% PEG 3350, 0.2 *M* KNO_3_ pH 6.9, while rgyr_Y851F (at 165 µ*M*) crystallized from 0.1 *M* HEPES–NaOH pH 7.0, 30% Jeffamine ED-2001. The rgyr_minlatch crystals were directly vitrified by hyperquenching (Warkentin & Thorne, 2007 ▸) in liquid nitrogen and the rgyr_Y851F crystals were cryoprotected with 10% ethylene glycol before plunging them into liquid nitrogen. Data were collected on beamline PX-II at Swiss Light Source using an EIGER 16M detector with X-rays of 1 Å wavelength and 0.2° oscillations. Intensities were integrated with *XDS* (Kabsch, 2010 ▸), scaled with *AIMLESS* and treated for anisotropy using *STARANISO* (Global Phasing). The rgyr_minlatch data were collected from a single crystal. For rgyr_Y851F, three data sets were merged to arrive at the final statistics reported in Table 1 ▸. The rgyr_Y851F data are roughly (within a 2% difference in unit-cell dimensions) isomorphous to the wild-type reverse gyrase structure with PDB entry 4ddt. High-resolution limits for the data were selected based on *I*/σ(*I*) ≥ 1 and CC_1/2_ ≥ 0.3 in the outer shell. Phases were generated by molecular replacement with *Phaser* (McCoy *et al.*, 2007 ▸). The previously determined wild-type structure (PDB entry 4ddu; Rudolph *et al.*, 2013 ▸) served as a search model for rgyr_Y851F, and PDB entry 4ddu without the latch domain was used for rgyr_minlatch. Models were rebuilt in *Coot* (Casañal *et al.*, 2020 ▸) and refined with *Phenix* (Liebschner *et al.*, 2019 ▸) using individual and TLS protocols for *B* values. The weights for *B* values, protein geometry and bulk-solvent mask were optimized automatically during refinement. The final structures contain a single molecule per asymmetric unit. Data-collection and refinement statistics were calculated using *phenix.table_one* (Table 1 ▸) with standard distributions for Ramachandran and *MolProbity* assessment taken from the Top8000 database of high-resolution protein structures. *AlphaFold*2 (AF2) models (Jumper *et al.*, 2021 ▸) were retrieved as version 4 from https://alphafold.ebi.ac.uk using a *PyMOL* plugin and colored according to their pLDDT values in the *B*-value column using the rainbow_rev palette in *PyMOL* (Schrödinger). *PyMOL* was also used to superimpose coordinates and render figures. Surface electrostatic potentials were calculated with *APBS* (Jurrus *et al.*, 2018 ▸).

### 2.3. Sequence analyses

A set of 184 unique reverse gyrase sequences from eubacteria was extracted from UniProt (UniProt Consortium, 2008 ▸, 2019 ▸). *ClustalW* and *ClustalW*2 from the *EMBOSS* suite (Madeira *et al.*, 2022 ▸) were used to generate the multiple sequence alignment and the phylogenetic tree, respectively. The sequences corresponding to the latch domains were extracted and analyzed for charge, hydrophobicity index and length. The charge at pH 7 was approximated by adding the number of Arg/Lys residues and half the number of His residues (p*K*_a_ ≃ 6.5) and subtracting the number of Glu/Asp residues. Pairwise alignment and identity/similarity assignment was performed using the Smith–Waterman algorithm as implemented in *EMBOSS* (Madeira *et al.*, 2022 ▸).

## 3. Results and discussion

### 3.1. Structure of the catalytically inactive Y851F variant of reverse gyrase

Cleavage-deficient variants of topoisomerases in which the catalytic tyrosine is replaced by a phenylalanine are widely used in topoisomerase research. Crystal structures of two type IA topoisomerases, *E. coli* topoisomerase III (Changela *et al.*, 2001 ▸) and *Sulfolobus solfataricus* topoisomerase III (PDB entries 6k8n and 6k8o; H. Q. Wang, J. H. Zhang, X. Zheng, Z. F. Zheng, Y. H. Dong, L. Huang & Y. Gong, unpublished work), reported little effect of this mutation on the local structure. The corresponding cleavage-deficient variant of reverse gyrase has also been used (Rodríguez, 2002 ▸), but no information on the structural changes associated with this mutation is available. In parallel to the structure-determination efforts of *T. maritima* reverse gyrase with a minimal latch, we also determined the crystal structure of the rgyr_Y851F variant (Fig. 1 ▸ *b*, Table 1 ▸). As expected, the overall structure of rgyr_Y851F is conserved and it superimposes on wild-type reverse gyrase with r.m.s.d.s of 1.3 Å (PDB entry 4ddu) and 0.56 Å (PDB entry 4ddt), respectively. The smaller r.m.s.d. when comparing rgyr_Y851F with PDB entry 4ddt may stem from the fact that these crystals are isomorphous (both belong to space group *C*2 with similar unit-cell dimensions). There are also few conformational differences between wild-type reverse gyrase and the Y851F variant around the catalytic site (Fig. 1 ▸ *b*). The Tyr/Phe residue at position 851 stacks on Pro660 and is further encased by the side chains of Asp668, Phe844, His852 and Arg853. A hydrogen bond present in the wild-type structures (PDB entries 4ddu and 4ddt) between the Tyr851 hydroxyl and Arg853 guanidinium groups is lost upon the Tyr851Phe change (Fig. 1 ▸ *b*). In all structures the guanidinium group of Arg853 remains fixed through a charged hydrogen bond to Asp670, but the aliphatic part of Arg853 can adopt slightly different rotamers. These minor structural differences are thus likely to be a result of inherent domain mobility in reverse gyrase. In summary, the Y851F variant adopts the same overall structure as wild-type reverse gyrase, and to­gether with the two wild-type *T. maritima* reverse gyrase crystal structures (PDB entries 4ddu and 4ddt) adds an independent data point for comparison with the structures and models of rgyr_minlatch and other reverse gyrases (see below).

### 3.2. The minimal latch forms a β-bulge loop connecting the helicase and topoisomerase domains

**Figure 2:**
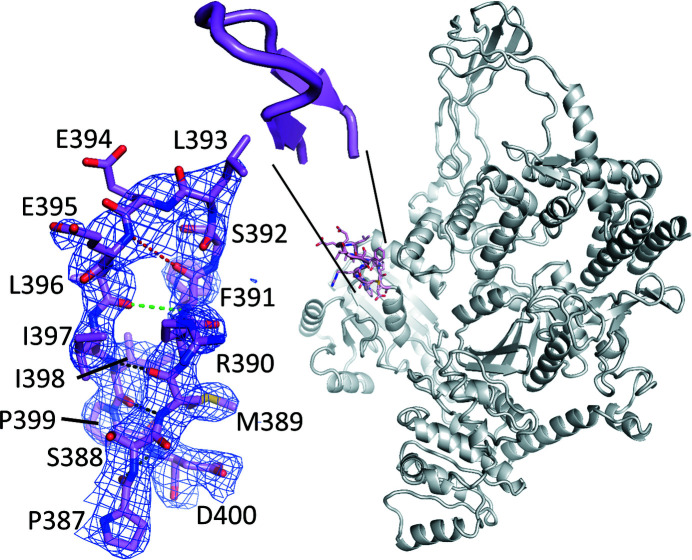
Crystal structure of *T. maritima* reverse gyrase with a minimal latch. The right-hand side shows the backbone of rgyr_minlatch with the minimal latch highlighted as a stick model (pink) with sequence 387-PSMR**FSLEEL**IIPD-400, with residues at the beginning and end of the latch that are conserved across reverse gyrases underlined and those forming the bulge loop in bold. Glu395–Asp400 in rgyr_minlatch corresponds to Glu456–Asp461 in authentic reverse gyrase. Zooming in on the minimal latch (cartoon depiction) shows that the sequence adopts a left-handed bulge loop inserted at the end of a short, two-stranded β-sheet. The 2*F*_o_ − *F*_c_ electron density on the left is contoured at 1 r.m.s.d. β-Sheet hydrogen bonds are shown as black dashed lines. The dashed green line is the first hydrogen bond (*i*, *i* + 5) characteristic of a type 2 β-bulge loop. The second characteristic hydrogen bond (*i*, *i* + 4), however, is absent (distance of 5 Å, dashed red line).

It has been shown that the globular part (residues 395–455) of the latch domain of *T. maritima* reverse gyrase is dispensable for positive supercoiling of DNA (Collin *et al.*, 2020 ▸), whereas deletion of the entire latch domain (residues 389–459, including the small β-sheet) abrogates supercoiling activity (Ganguly *et al.*, 2013 ▸). To gain insight into the structural basis for this differential effect on activity, we determined the crystal structure of rgyr_minlatch. Although rgyr_minlatch crystallized in a different space group, the overall structure (Fig. 2 ▸) is similar to the structure of the full-length enzyme (Fig. 1 ▸ *a*), revealing a padlock shape. Superposition of all residues except the latch region in rgyr_minlatch results in r.m.s.d. values of 0.94 Å (PDB entry 4ddu), 0.98 Å (PDB entry 4ddt) and 1.0 Å (rgyr_Y851F). Equally small r.m.s.d. values are observed for the individual helicase domains (0.94–0.98 Å) and topoisomerase domains (0.52–0.67 Å), demonstrating that deletion of the globular domain of the *T. maritima* reverse gyrase latch has no significant effect on the reverse gyrase structure outside the latch.

The minimal latch formed by residues 387-PSMR**FSLEELII**PD-400 emanates from the H2 sub­domain and retains its short β-sheet, *i.e.* the first and last four residues of the sequence. The β-sheet is topped by a loop of six residues of sequence FSLEEL generated by deletion of the region 395–455 (Δ in the sequence; Fig. 2 ▸). The hydrogen-bonding characteristics of the loop resemble those of a type 2 β-bulge loop (Sibanda *et al.*, 1989 ▸; Milner-White, 1987 ▸). Whereas in standard β-bulges additional residues that break the hydrogen-bonding pattern are inserted into a strand of a β-sheet, the inserted residues are located at the end of an antiparallel β-sheet in β-bulge loops. In contrast to β-turns, which have a length of four residues, the loops in β-bulges consist of five or six residues, distinguished as type 1 and type 2, respectively. Typical of type 2 β-bulge loops, the minimal latch in rgyr_minlatch forms a hydrogen bond between the NH group of residue *i* (Phe391) and the carbonyl group of residue *i* + 5 (Leu396, green in Fig. 2 ▸). A second characteristic of type 2 β-bulge loops, a hydrogen bond between the carbonyl group of residue *i* and the NH group of residue *i* + 4 (Glu395), is not present, however (distance of 5 Å; red in Fig. 2 ▸). Nonetheless, the conformational variability in type 2 β-bulge loops is large (Sibanda *et al.*, 1989 ▸) and we keep this term for the minimal latch here.

**Figure 3:**
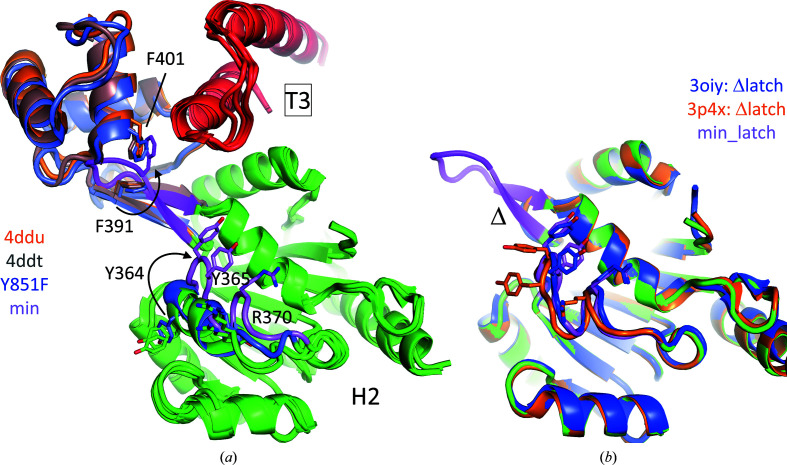
Latch–T3 interface and latch-associated conformational changes in *T. maritima* reverse gyrase. Structures were superposed onto their H2 domains lacking the latch regions. (*a*) Cutout of the H2 domain (green), latch regions and the T3 part (red) of the topoisomerase domain. The twisted β-sheet in rgyr_minlatch is colored magenta. An arrow indicates the movement of Phe391 in the β-bulge loop to a location previously occupied by Phe401 of the authentic latch domain. An α-helix (green for wild type and blue for Y851F) rearranges into a loop structure in rgyr_minlatch (magenta). The largest movement is apparent for Tyr364, which flips to the other side (indicated by an arrow). (*b*) The conformational change in H2 is not due to the β-bulge loop, since it is also observed in structures of the helicase domain with the entire latch removed (indicated by Δ).

Compared with the wild-type and Y851F reverse gyrase structures, the β-sheet in rgyr_minlatch is twisted towards the T3 region of the topoisomerase domain (Fig. 3 ▸ *a*). The twist is a result of Phe391, the first residue in the β-bulge loop, rotating its side chain into a void that is normally occupied by Phe401. Phe401 is one of the few residues in the latch that engages in direct contacts with the T3 region of the topoisomerase domain. Deletion of the globular domain entails the removal of Phe401, apparently prompting Phe391 to fill the void and maintain van der Waals interactions with the methylene groups of Glu850 in the T3 region of the topoisomerase domain. Notably, the latch domains in *T. maritima* and *A. fulgidus* reverse gyrases form only few contacts with their topoisomerase domains, and the inter-domain interfaces are small and rather shallow. Both aspects argue in favor of a transient interaction of latch and topoisomerase domains, in accord with the proposed unlatching and opening of the topoisomerase domain during supercoiling (Rodríguez & Stock, 2002 ▸; Lulchev & Klostermeier, 2014 ▸). In rgyr_minlatch, the paucity of interactions between the remaining latch and the topoisomerase domain is extreme. Apart from Ile398 in the β-sheet, the location of which is the same in all *T. maritima* structures, Phe391 is the only residue that makes any interaction with the topoisomerase domain. In conclusion, it seems that the minimal latch acts as a steric block or placeholder that allows the helicase and topoisomerase domains to reversibly touch each other.

**Figure 4:**
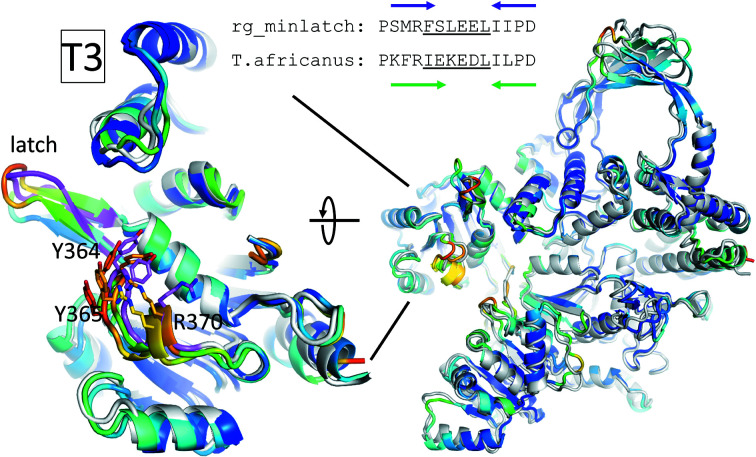
Comparison of *T. maritima* rgyr_minlatch with a natural minimal latch in *T. africanus* reverse gyrase. The AF2 models of UniProt ID B7IEV8 (UniProt Consortium, 2008 ▸, 2019 ▸) and rgyr_minlatch are shown on the right, superimposed on the crystal structure of rgyr_minlatch in gray. The backbone of the AF2 model is colored according to the confidence of the prediction, described as the predicted Local Distance Difference Test (pLDDT) as a percentage. Here, pLDDT ranges from 51% to 98% and is colored with the ‘rainbow_rev’ palette in *PyMOL*. Dark blue areas have pLDDT >90%, green 90–70%, yellow 70–50% and red <50%. On the left-hand side, the H2/latch regions are enlarged and rotated by 90° around the *x* axis. The sequences of the latch regions are given together with the strands forming the two-stranded β-sheet, which is slightly larger (green arrow) in the predicted *T. africanus* reverse gyrase. The conformational change observed in rgyr_minlatch is highlighted in pink, with key side chains shown as stick models. The same conformation is predicted in the *T. africanus* and rgyr_minlatch models, as well as in the AF2 models of *A. fulgidus*, *T. tengcongensis* and *S. solfataricus* reverse gyrases (Supplementary Fig. S1).

Outside the latch region, a notable difference between rgyr_minlatch and other *T. maritima* reverse gyrase structures is the unfolding of an α-helix (sequence QAYYGKLTRGVD) in subdomain H2 (Fig. 3 ▸). This region has elevated *B* values compared with the mean of the H2 domain, indicating a proneness to plasticity. The α-helix is present in both wild-type structures but is in a slightly different conformation in the Y851F structure. The backbone trace of rgyr_minlatch is shifted by 7–8 Å (C^α^ atoms of Tyr364, Tyr365 and Arg370) compared with wild-type reverse gyrase, with the hydroxyl groups of Tyr364 shifted apart by 21 Å (Fig. 3 ▸ *a*). Although it appears to be conceivable that this conformational difference may be due to the nearby β-bulge loop, this is not the case: the same conformation of this α-helix has been observed in two independent crystal structures of the *T. maritima* helicase domain lacking the entire latch domain (Ganguly *et al.*, 2011 ▸; Fig. 3 ▸ *b*; PDB entries 3oiy and 3p4x). These structures show the same main-chain trace as in rgyr_minlatch, although the rotamers of the Tyr364, Tyr365 and Arg370 side chains are different. These side-chain conformations are not imposed by crystal-packing effects. Hence, this conformational difference could be due to the reduced mass or volume of the latch in rgyr_minlatch compared with authentic reverse gyrases. For most of the ∼10^8^ sequences in the UNIREF90 database (Suzek *et al.*, 2015 ▸), *AlphaFold*2 (AF2) models have now been predicted (Jumper *et al.*, 2021 ▸), including several reverse gyrases. Notably, in a number of AF2 models of reverse gyrases with sizeable latch domains the same main-chain trace is predicted, among them the reverse gyrases from *Archaeo­globus fulgidus*, *Thermoanaerobacter tengcongensis*, *Sulfolobus solfataricus* and *Thermosipho africanus* (left in Fig. 4 ▸; Supplementary Fig. S1). Of these models, *T. africanus* reverse gyrase is particularly interesting since it has a natural minimal latch.

### 3.3. *T. africanus* reverse gyrase: an enzyme with a natural minimal latch

Reverse gyrase from *T. africanus* is an example of a naturally occurring reverse gyrase that contains a small latch of sequence PKFRIEKEDLILPD with the same number of residues as rgyr_minlatch. We had previously hypothesized that this region forms a β-hairpin that acts as a minimal latch, similar to the β-bulge loop in *T. maritima* reverse gyrase (Collin *et al.*, 2020 ▸; Lulchev & Klostermeier, 2014 ▸). We compared the *T. maritima* rgyr_minlatch crystal structure with the model of *T. africanus* reverse gyrase. Since the AF2 model of *T. africanus* was predicted before the rgyr_minlatch coordinates were deposited in the PDB, direct model bias from rgyr_minlatch in the training set is excluded. Nevertheless, we note that these structural model predictions must be interpreted with care.

The *T. africanus* AF2 model retains the overall architecture established by the reverse gyrases from *A. fulgidus* and *T. maritima*. The AF2 model and the rgyr_minlatch crystal structure superimpose with an r.m.s.d. of 1.3 Å, despite a sequence identity and similarity of only 54% and 73%, respectively (Fig. 4 ▸, right). Further, the latch region in *T. africanus* reverse gyrase is predicted to form a true, non-twisted β-hairpin with a terminal *i*, *i* + 4 hydrogen bond but similar overall to the β-bulge loop in *T. maritima* rgyr_minlatch (Fig. 4 ▸, left). Interestingly, no interactions are predicted for the β-hairpin latch of *T. africanus* reverse gyrase. The pLDDT values for the *T. africanus* latch are between 50% and 70%, classifying the confidence of the prediction in this region as moderate. It is possible that AF2 may have optimized the *T. africanus* latch region to match the canonical protein geometry of an untwisted β-hairpin present in its training models, leaving the possibility that *T. africanus* reverse gyrase adopts a similarly tilted latch to that present in rgyr_minlatch.

There are three crucial differences in the minimal latch regions between the crystal structure of *T. maritima* reverse gyrase and the AF2 model of *T. africanus* reverse gyrase that influence its interaction with the topoisomerase domain: (i) the Phe(391)-to-Ile(388) change, (ii) the bulge in *T. maritima* reverse gyrase, which is a β-hairpin in the *T. africanus* enzyme, and (iii) a tilt of the *T. africanus* latch away from the topo­isomerase domain. The bulge, the larger side chain and the closer proximity of the latch to the topoisomerase domain in *T. maritima* reverse gyrase jointly enable an interaction between the latch and the topoisomerase domain. All of these features are absent in the *T. africanus* AF2 model: the corresponding side chain is smaller, the bulge is absent and the loop is tilted away from the topoisomerase domain.

Interestingly, when we generated an AF2 model of *T. maritima* rgyr_minlatch the β-bulge structure was predicted to be in a conformation similar to *T. africanus* reverse gyrase, with no interaction of Phe391 with the T3 domain (Fig. 4 ▸). This argues that the differences between the experimental *T. maritima* rgyr_minlatch structure and *T. africanus* reverse gyrase reflects a limitation of AF2 in predicting conformations without precedent in the PDB. Overall, it seems that the task of coupling the helicase domain to the topoisomerase domain can be achieved through many different structural solutions.

### 3.4. Latch domains in reverse gyrases are steric blocks that are flexibly attached to topoisomerase domains

**Figure 5:**
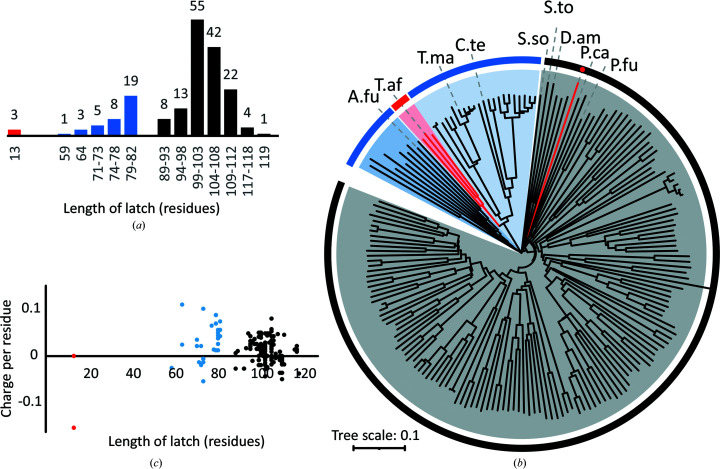
Properties of the reverse gyrase latch regions. (*a*) Length distribution. The horizontal axis is given in bin widths of five residues. A single number or a smaller bin width is stated if the actual sequence lengths in that bin were narrower than the bin width (for example the first cluster has only three sequences, all of which comprise 13 residues and belong to *Thermosipho*). The number on top of the bars states the number of members per bin. The lengths of the latch regions of 184 reverse gyrases fall into three clusters, which are colored differently. (*b*) Phylogenetic tree of 184 reverse gyrases. The latch length clusters, highlighted in red (13 residues), blue (59–82) and black (89–119), are also clustered when reverse gyrases are grouped in terms of evolutionary relationship. The single exception in this set, marked with a red dot, is reverse gyrase from an as yet unclassified Thermotogae bacterium with a latch length of only 59 residues. The three main clades of the tree are color-coded in gray (1), light blue (2) and blue (3). The branch within clade 2 containing *T. africanus* reverse gyrase is highlighted in red. The reverse gyrases that were selected for further structural analysis (Fig. 6 ▸) are labelled. A.fu., *A. fulgidus*; C.te., *Caldanaerobacter subterraneus* subsp. *tengcongensis*; D.am., *Desulfurococcus amylolyticus*; P.ca., *Pyrobaculum calidifontis*; P.fu., *Pyrococcus furiosus;* S.so., *Saccharolobus solfataricus*; S.to., *Sulfurisphaera tokodaii*; T.af., *Thermosipho africanus*; T.ma., *T. maritima*. An annotated version of the tree is given in Supplementary Fig. S3. (*c*) Electrostatics. The overall charge of the latch at neutral pH, calculated as stated in Section 2 and divided by the length of the latch, is plotted against latch length. Almost two thirds (62%) of the latch regions have an overall positive charge. The red data points (two at zero and one at −0.15) belong to the three *Thermosipho* sequences of only 13 residues.

**Figure 6:**
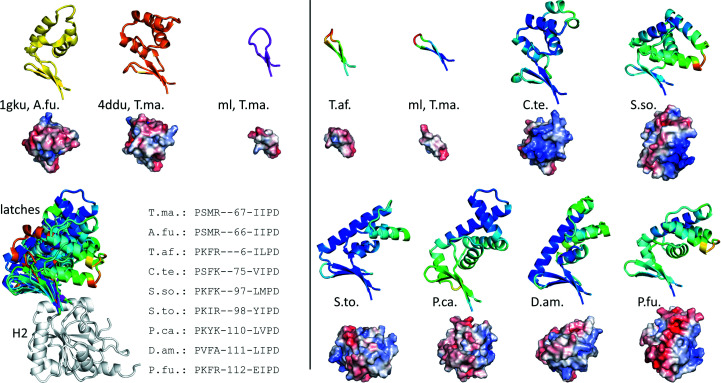
The different shapes, sizes and electrostatic surface potentials of latch domains. The vertical line separates the AF2 models (right) from the crystallographic structures (left). A superposition of all structures and models on their H2 domains is shown at the lower left. The sequences of the two β-­hairpin-forming β-strands are given with the number of residues forming the rest of the latch in between. Ribbon representations are to scale and in the same orientation as in the superposition. Surfaces of latch domains are rotated by 90° about the *x* axis to provide a top view of the latch with the right-hand side pointing towards the T3 region of the topoisomerase domains. A region of positive electrostatic potential is apparent on the right-hand side of several, but not all, latch domains. Crystal structures are A.fu, *A. fulgidus*; T.ma., *T. maritima*; ml, rgyr_minlatch from *T. maritima*. AF2 models are T.af., *T. africanus* (UniProt B7IEV8); ml, T.ma., rgyr_minlatch; C.te., *C. subterraneus* subsp. *tengcongensis* (Q8R979); S.so., *S. solfataricus* (Q97ZZ8); S.to., *S. tokodaii* (Q971T7); P.ca., *P. calidifontis* (A3MU01); D.am., *D. amylolyticus* (B8D628); P.fu., *P. furiosus* (P95479).

We next asked whether there is a common denominator from a structural perspective in how the different latches modulate inter-domain communication in reverse gyrases. To address this question, we first generated a multiple sequence alignment of 184 non-identical reverse gyrase sequences from eubacteria. For all of these sequences, the latch regions were defined based on the sequence positions of the first and last residues forming the latch in the crystal structures available. The lengths of these latch regions fall into three clusters. Three sequences, all from *Thermosipho*, comprise only 13 residues, 36 sequences have 59–82 residues and 145 sequences have 89–119 residues (Fig. 5 ▸ *a*). In a phylogenetic tree generated from all 184 complete sequences, reverse gyrases are clearly grouped according to the lengths of their latch regions (Fig. 5 ▸ *b*). The phylogenetic tree places reverse gyrases into three main clades rooted at the origin. Clade 1 includes the majority of all reverse gyrases analyzed, all of them except one with long latches, supporting an evolutionary relationship of reverse gyrases with long latch regions. Clades 2 and 3 comprise reverse gyrases with latches of intermediate lengths. Rooted at (or at least near) the origin, the latch regions of intermediate lengths might stem from a common evolutionary ancestor. Interestingly, the reverse gyrases from *T. maritima* and *A. fulgidus* are located in evolutionarily distant clades 2 and 3, respectively, which may rationalize the different structures of the globular domains of their latches despite their similar lengths (Fig. 6 ▸, left). The branch leading to *Thermosipho* reverse gyrases with the shortest latch is located within clade 2 of enzymes with intermediate latch lengths, which indicates that *Thermosipho* might have ‘lost’ its globular latch domain during evolution.

Next, we selected representatives of each cluster (see Fig. 5 ▸ *b*) and superimposed the corresponding AF2 models on the H2 subdomain of *T. maritima* reverse gyrase (PDB entry 4ddu; Fig. 6 ▸). The small β-sheet emanating from the H2 domain is conserved across all reverse gyrase structures and models. In contrast, the latch regions differ greatly in both size and predicted structure. While the smallest number of residues emanating from the base of the latch (six) inevitably amounts to a β-hairpin-like structure, the number of residues is not a general predictor for the structure that the latch adopts. The latch domains in the crystal structures of *T. maritima* and *A. fulgidus* have 67 and 66 residues, respectively, but fold into quite different globular domains that cannot sensibly be superimposed. Both the *A. fulgidus* and *T. maritima* latches have a three-stranded β-sheet at their base, in which a small additional β-strand extends the β-bulge loop. In fact, all AF2 models of reverse gyrases considered here, except for that from *T. africanus*, have a three- to four-stranded β-sheet at their base that serves as a platform for α-helical domains that can be either globular or extended (Fig. 6 ▸). Only the largest latches seem to share a common structural feature: an overall L-shape with a long central α-helix flanked by 3–4 shorter α-helices extending at an angle of approximately 90° from the β-sheet at the base. These latch domains are large enough to contact the insert regions in H1 (cyan for *T. maritima* in Fig. 1 ▸; Supplementary Fig. S2), raising the possibility that these insertions in the two helicase domains communicate with each other during supercoiling. Even evolutionarily very distant reverse gyrase sequences are predicted to fold into such an L-shaped latch (models are not shown, but see Supplementary Fig. S3).

Since the latch interacts with DNA during the catalytic cycle (del Toro Duany *et al.*, 2014 ▸; Ganguly *et al.*, 2011 ▸; Collin *et al.*, 2020 ▸), we next turned to electrostatic surface potentials as a possible common feature among reverse gyrase latches. To this end, we used the available crystal structures and the AF2 models. We reasoned that the coordinate accuracy of these models justifies general conclusions on their surface properties. This is supported by the fact that AF2 models are often accurate enough to serve as models in molecular replacement (Simpkin *et al.*, 2022 ▸; McCoy *et al.*, 2022 ▸). In all AF2 models except that from *T. africanus*, a trend to positive potential is visible for the part of the latch pointing towards the T3 region of the topoisomerase domain, which might contribute to interactions with DNA (Fig. 6 ▸). This positive patch is also present in the *T. maritima* reverse gyrase crystal structure but not in the *A. fulgidus* enzyme or in rgyr_minlatch. An estimation of the overall charge at neutral pH for all 184 reverse gyrase latch sequences reveals the presence of a positive potential for the latch, although there are a few exceptions. About two thirds of all sequences (114) are predicted to have an overall positive charge, 13 are electroneutral and 57 are slightly negatively charged (Fig. 5 ▸ *c*). Thus, latch regions might depend on other mechanisms or other protein regions for inter-domain communication and/or DNA binding. It is possible that the minimal function of the latch is to act as a small, steric block. Additionally, larger patches of positive electrostatic potential and specific interactions with the topoisomerase domain and with other regions of the enzyme enable varying degrees of coupling throughout the catalytic cycle.

## 4. Conclusions

In this study, we provide evidence that the cleavage-deficient Y851F variant of *T. maritima* reverse gyrase shares many structural characteristics with the wild-type enzyme. We also show that the engineered and functional minimal latch of a reverse gyrase folds into a modified β-hairpin structure known as a β-bulge loop that has precedence in a naturally occurring enzyme. From the three latch structures currently determined by crystallography, it becomes clear that the size and shape of this domain can vary greatly. This notion is further supported by AF2 models, in which the latches appear to be so versatile in both structure and electrostatic properties that the only common denominator for latch function is by way of a steric block between the helicase and topoisomerase domains. Regions with various degrees of positive electrostatic potential enable interactions with DNA, and specific interactions formed between the latch and the lid provide varying degrees of coupling between the helicase and topoisomerase domains. The versatility of the latches thus suggests multiple modes of action in supercoiling that may employ different regions of reverse gyrase during the catalytic cycle (Rodríguez, 2002 ▸). Analysis of crystal structures and comparison with AF2 models reveals a recurrent conformational change in the H2 domain. Interestingly, uncovering this conformational change only became possible when part of or the entire latch was deleted. It is conceivable that this conformational change has a biological role and occurs upon movement of the latch during the supercoiling reaction. While AF2 models have proven to be helpful, the current data situation is limited: only a few crystal structures are available and a DNA–reverse gyrase complex is lacking. A better understanding of both the functioning of the latch domain and the role of conformational changes in the H2 domain during supercoiling requires structural information on reverse gyrase–DNA complexes at different stages of the catalytic cycle.

*Note added in proof.* During proofreading of this article a higher resolution dataset (2.4 Å) in a different space group (*P*6_1_22) for reverse gyrase with a minimal latch was collected that is congruent with all salient features of the data described in the paper. The refined coordinates and structure factors have been deposited with the PDB with ID 8ofb.

## Supplementary Material

PDB reference: reverse gyrase with a minimal latch, 7fse

PDB reference: Y851F mutant, 7fsf

Supplementary Figures. DOI: 10.1107/S2059798323002565/rr5231sup1.pdf

## Figures and Tables

**Table 1:** Data-collection and refinement statistics Values in square brackets and parentheses correspond to the lowest and highest resolution shells, respectively. *R* values and CC_1/2_ are defined in Diederichs & Karplus (1997 ▸) and Karplus & Diederichs (2012 ▸), respectively.

	Rgyr_minlatch	Rgyr_Y851F
PDB code	7fse	7fsf
Detector	EIGER2 16M	EIGER2 16M
Wavelength (Å)	1	1
Rotation range (°)	200	200
Oscillation (°)	0.1	0.1
Resolution range (Å)	[88.3–9.0] 88.3–2.89 (3.12–2.89)	[87.0–8.5] 51.9–2.77 (2.87–2.77)
Space group	*P*4_1_2_1_2	*C*2
*a*, *b*, *c* (Å)	91.8, 91.8, 323.3	183.9, 103.3, 95.7
α, β, γ (°)	90, 90, 90	90, 116.8, 90
Total reflections	[15948] 370664 (19937)	[16017] 337743 (16550)
Unique reflections	[1249] 25024 (1252)	[1481] 29621 (1482)
Multiplicity	[12.8] 14.8 (15.9)	[10.8] 11.4 (11.2)
Completeness (%)	[99.8] 92.8 (49.8)	[99.8] 92.8 (59.3)
Mean *I*/σ(*I*)	[18.8] 7.3 (1.4)	[17.4] 6.4 (1.3)
Wilson *B* factor (Å^2^)	66.2	66.3
*R*_merge_	[0.092] 0.387 (4.66)	[0.107] 0.288 (1.98)
*R*_meas_	[0.096] 0.401 (4.80)	[0.113] 0.366 (2.08)
*R*_p.i.m._	[0.033] 0.104 (1.19)	[0.035] 0.090 (0.86)
CC_1/2_	[0.993] 0.994 (0.540)	[0.993] 0.993 (0.393)
Reflections used		
In refinement	25016 (311)	29616 (262)
For *R*_free_	1231 (15)	1475 (15)
*R*_work_	0.2123 (0.3380)	0.2191 (0.3450)
*R*_free_	0.2639 (0.3143)	0.2733 (0.4825)
CC(work)	0.923 (0.627)	0.919 (0.665)
CC(free)	0.910 (0.777)	0.731 (0.618)
No. of atoms		
Protein	8521	9029
Ligand	23	2
Solvent	17	0
No. of protein residues	1040	1102
R.m.s.d.		
Bond lengths (Å)	0.002	0.003
Angles (°)	0.43	0.51
Ramachandran statistics		
Favoured (%)	97.7	96.8
Allowed (%)	2.3	3.2
Rotamer outliers (%)	2.1	3.3
Clashscore	6.5	5.2
Average *B* factor (Å^2^)		
Overall	71.8	77.8
Protein	71.9	77.8
Ligand	71.0	74.9
Solvent	43.6	—
No. of TLS groups	4	8
