# Supplementary material for: Structure of reverse gyrase with a minimal latch that supports ATP-dependent positive supercoiling without specific interactions with the topoisomerase domain
Source: Acta Crystallogr D Struct Biol. 2023 May 19;79(Pt 6):498–507. doi: 10.1107/S2059798323002565 (PMC10233626; doi:10.1107/S2059798323002565)
Supplement: Supplementary file 1 [file d-79-00498-sup1.pdf]

STRUCTURAL BIOLOGY

Volume 79 (2023)

Supporting information for article:

Structure of reverse gyrase with a minimal latch that supports ATP-dependent positive supercoiling without specific interactions with the topoisomerase domain

Vaibhav Mhaindarkar, René Rasche, Daniel Kümmel, Markus G. Rudolph and Dagmar Klostermeier

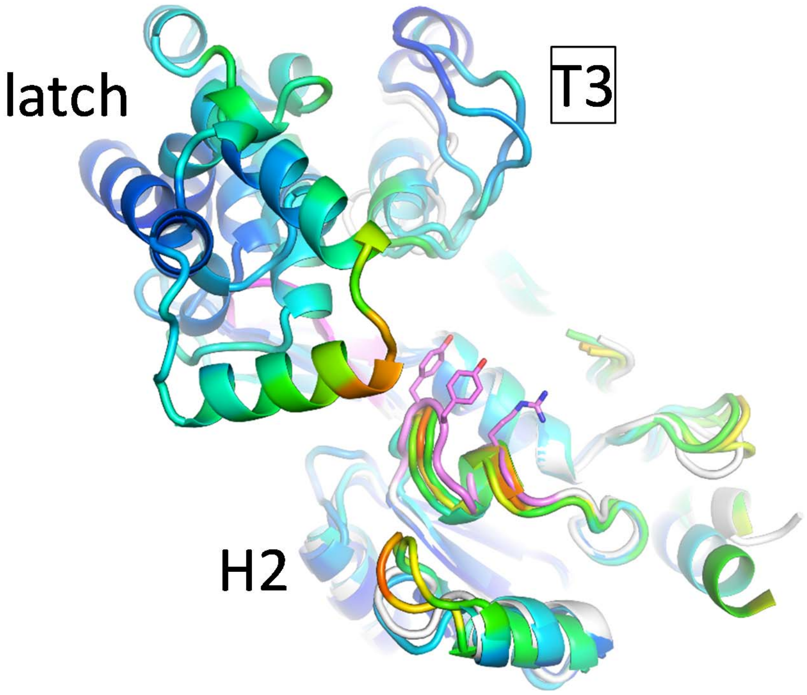

Figure S1 AF2 models with the same conformations in H2 as rgyr_minlatch. Shown are the AF2 models of A. fulgidus, S. solfataricus, and C. subterraneus subsp. Tengcongensis (also known as Thermoanaerobacter tengcongensis) reverse gyrases. The color code is the same as in Fig. 4. Other AF2 models of reverse gyrase sequences have different conformations, but the repeated occurrence of this exact backbone trace hints towards a stable conformation that might be of biological significance.

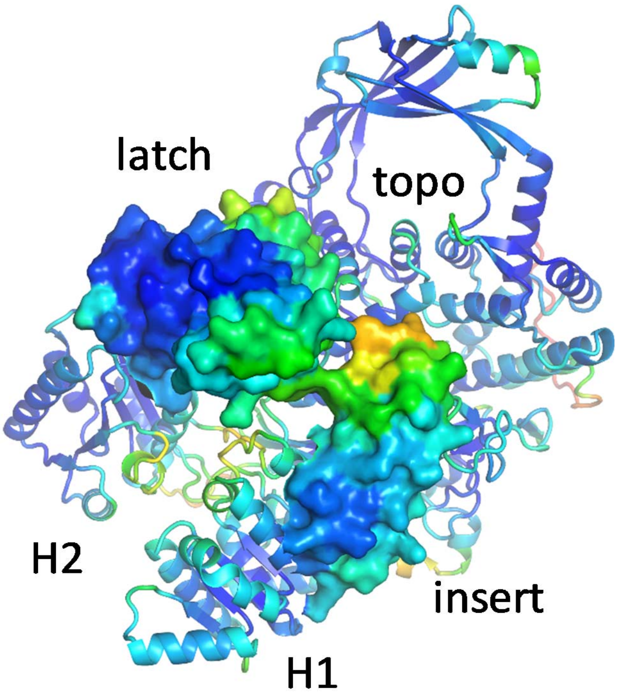

Figure S2 AF2 model of *D. amylolyticus* reverse gyrase.The AF2 model of *D. amylolyticus* reverse gyrase (UniProt B8D628) has such large latch and insert regions that they are predicted to touch each other. Both regions are shown as surface representation. In addition, this is one of several examples where the H1 and H2 subdomains come very close to each other, mimicking the closed form of the helicase domain. Others are UniProt I3XTC8, also from *D. amylolyticus* and A3MU01 from *P. calidifontis*.

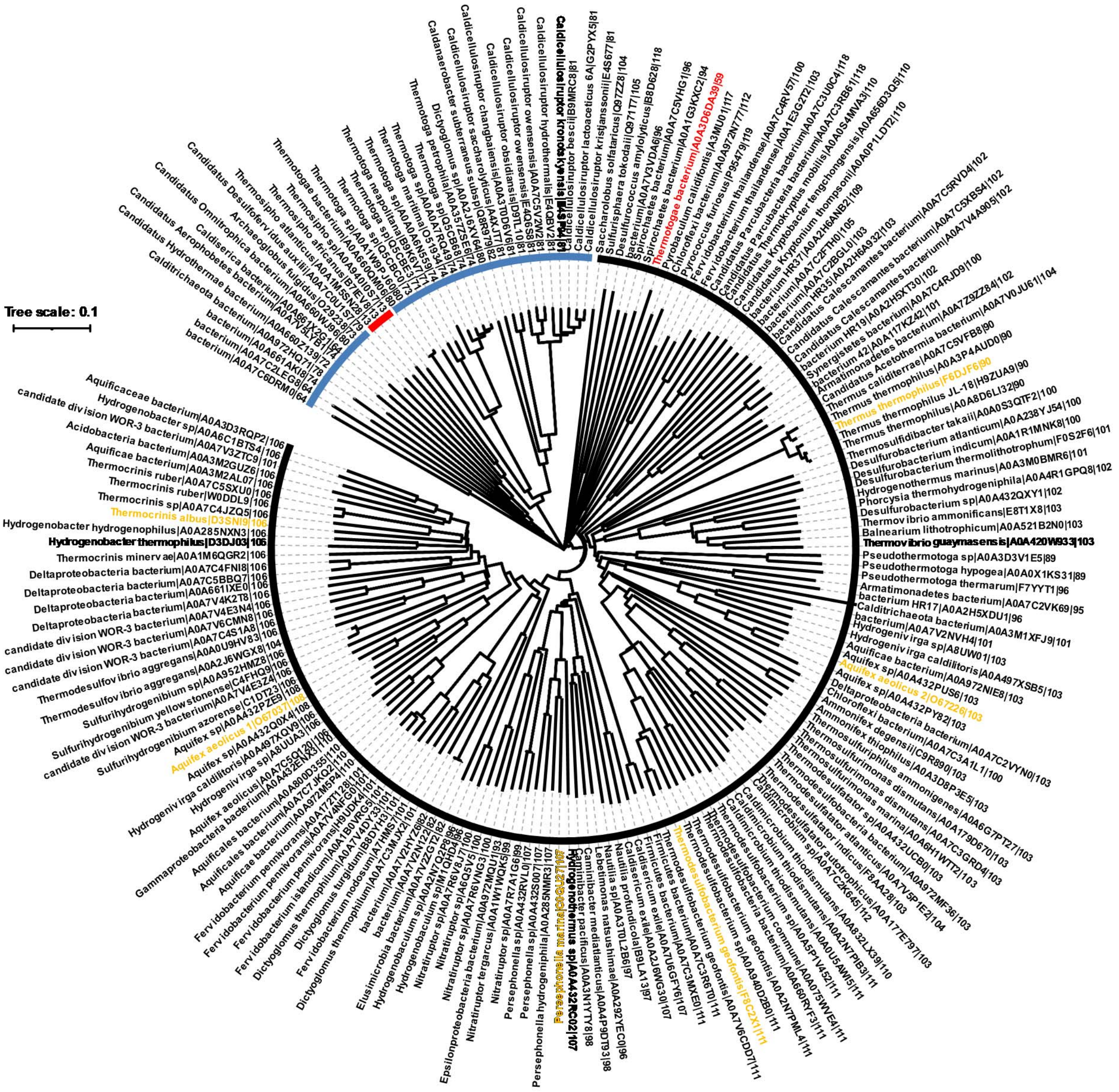

Figure S3 Phylogenetic tree of the 184 reverse gyrase sequences discussed in the main text. The annotation is: organism name | UniProt ID | length of the latch. Sequences highlighted in yellow were selected based on their large evolutionary distance to the ones discussed in the text. The corresponding AF2 models show the same L-shape for the latch domains (not shown) as displayed in Fig.6, indicating that the cluster of reverse gyrases with the largest latch domains bears structural similarity throughout evolution.
